# Genetic Variability of Methane Production and Concentration Measured in the Breath of Polish Holstein-Friesian Cattle

**DOI:** 10.3390/ani11113175

**Published:** 2021-11-06

**Authors:** Mateusz Sypniewski, Tomasz Strabel, Marcin Pszczola

**Affiliations:** Department of Genetics and Animal Breeding, Poznan University of Life Sciences, 60-637 Poznan, Poland; mateusz.sypniewski@up.poznan.pl (M.S.); tomasz.strabel@up.poznan.pl (T.S.)

**Keywords:** methane emission, dairy cows, genome-wide association study

## Abstract

**Simple Summary:**

Methane is one of the main contributors to climate change. A potential to reduce emissions by genetic selection exists; however, the genetic architecture of methane production remains largely unknown. We aimed to estimate its heritability and to perform genome-wide association studies for the identification of candidate genes associated with daily methane production and concentration. Methane was measured in the air exhaled by dairy cows during milking in an automated milking system and was analyzed using genomic information that was available for some of the cows. We showed that methane production and concentration are partly controlled by genes; however, no major genes were found. The estimated heritabilities indicate that selection for lower methane emission could be successful.

**Abstract:**

The genetic architecture of methane (CH_4_) production remains largely unknown. We aimed to estimate its heritability and to perform genome-wide association studies (GWAS) for the identification of candidate genes associated with two phenotypes: CH_4_ in parts per million/day (CH_4_ ppm/d) and CH_4_ in grams/day (CH_4_ g/d). We studied 483 Polish Holstein-Friesian cows kept on two commercial farms in Poland. Measurements of CH_4_ and carbon dioxide (CO_2_) concentrations exhaled by cows during milking were obtained using gas analyzers installed in the automated milking system on the farms. Genomic analyses were performed using a single-step BLUP approach. The percentage of genetic variance explained by SNPs was calculated for each SNP separately and then for the windows of neighbouring SNPs. The heritability of CH_4_ ppm/d ranged from 0 to 0.14, with an average of 0.085. The heritability of CH_4_ g/d ranged from 0.13 to 0.26, with an average of 0.22. The GWAS detected potential candidate SNPs on BTA 14 which explained ~0.9% of genetic variance for CH_4_ ppm/d and ~1% of genetic variance for CH_4_ g/d. All identified SNPs were located in the TRPS1 gene. We showed that methane traits are partially controlled by genes; however, the detected SNPs explained only a small part of genetic variation—implying that both CH_4_ ppm/d and CH_4_ g/d are highly polygenic traits.

## 1. Introduction

Methane (CH_4_) is one of the main contributors to climate change. Its global warming potential over 100 years is 28 times higher than that of carbon dioxide (CO_2_) [1]. Among various sources of emissions, the livestock industry plays an important role [2]. Although CH_4_ does not accumulate over time, by reducing its emission the livestock industry can slow down the climate change process [3].

Among enteric methane mitigation strategies, which include nutritional and management strategies, selective breeding to reduce the amount of methane produced per kg of milk is of interest. Genetic reduction of CH_4_ emission requires measurements of emission from many animals. Emission of CH_4_ can be measured in several ways. One group of measuring methods is breath analyzing techniques. These are well-documented methods based on the principle that about 90% of enteric CH_4_ is released during eructation events and by breathing [4]. These methods are cost-effective and non-invasive. Some of them are based on infrared spectroscopy and have been used successfully by several research groups [5,6,7], showing the possibility of acquiring large amounts of data suitable for genetic analysis. 

Once the methane measurements are collected, one may define various methane phenotypes, which include: methane production, methane intensity, methane yield, or residual methane production [8]. Commonly, the amount of methane produced by an animal [9,10] is expressed in grams or liters of CH_4_ per day, which is calculated in accordance to Madsen et al. (2010) [11]. The aforementioned phenotype is based on the CH_4_/CO_2_ ratio (ppm) multiplied by CO_2_ L/day estimated from the heat production unit [11,12]. An alternative solution is to use raw CH_4_ concentrations, which are obtained without taking into account other traits. Thus far there have been few studies based on direct CH_4_ emission [13,14], and only the study of van Engelen et al. (2018) [15] provides heritability estimates—but without a focus on more detailed genetic architecture.

The genetic architecture of CH_4_ remains largely unknown. Several studies have attempted to approach this issue, and results indicate that genes control approximately 20–30% of methane emission variability [9,10,16]. Differences between the estimates may arise from the complexity of the emission process and differences between populations and periods of measurement. In addition, the emission process is affected by several factors, e.g., biological processes in the rumen, type of diet, amount of feed intake, and size of the animal [17].

In this paper, we aim to estimate heritability for two methane phenotypes: CH_4_ ppm/day (CH_4_ ppm/d) and CH_4_ grams/day (CH_4_ g/d), and to perform genome-wide association studies (GWAS) for the identification of candidate genes associated with CH_4_ ppm/d and CH_4_ g/d in the air exhaled by dairy cows. 

## 2. Materials and Methods

We studied 483 Polish Holstein-Friesian cows kept on two commercial farms in Poland. Detailed information on farms, measuring set-up, and data processing of records used in this study can be obtained from Pszczola et al. (2017) [7]. The CH_4_ and CO_2_ concentrations were measured on Farm 1 during two periods: from 02 December 2014 to 03 February 2016, and 01 June 2016 to 17 September 2016 from the 15th to 305th day in milk (DIM). Measurements on Farm 2 were taken over the period of 05 February 2016 to 14 March 2016, from the 5th to 305th DIM. Measurements of CH_4_ and CO_2_ concentrations were taken during milking using an FTIR gas analyzer (GASMET 4030; Gasmet Technologies Oy, Helsinki, Finland) that was installed in the feeding bin of the Automated Milking System (AMS, Astronaut, Lely Industries, NV, Maassluis, The Netherlands). Cows were milked repeatedly during the experiment. 

Measurements of CH_4_ and CO_2_ concentrations were corrected for diurnal variation with a Fourier series approach [18,19] using the model described by Pszczola et al. (2017) [7], and averaged per cow per day. Therefore, the analyzed phenotypes were daily average CH_4_ concentrations (CH_4_ ppm/d), with a total of 34,359 CH_4_ measurements. The detailed number of observations and average CH_4_ emission per farm, along with production parameters, are presented in Table 1. The cows had ad-libitum group access to partial mixed rations, and additional concentrates were given to cows during milking. Further details on the feeding regime can be obtained from Pszczola et al. (2017) [7].

CH_4_ production was calculated based on Madsen et al. (2010) [11] by multiplying the CH_4_ to CO_2_ concentration ratio by the expected CO_2_ production (liters/day) estimated from heat production [11,12]. This estimation of CO_2_ production used the milk production, live weight, and day of pregnancy obtained from the AMS system. This resulted in an estimated CH_4_ production expressed in liters per day, and was converted to grams per day using a CH_4_ density of 0.668 g/L, which assumes normal temperature (20 °C) and pressure (101.325 kPa). For more details, see Pszczola et al. (2017) [7].

Overall, 330 of the 483 cows were genotyped with the Illumina BovineSNP50 v2.0 BeadChip (Illumina Inc., San Diego, CA, USA). The SNP data were processed with the following quality control checks: (1) being in Hard–Weinberg equilibrium, (2) not being monomorphic, and (3) having an SNP call rate of above 0.95. We also removed SNPs located on the sex chromosomes and unassigned SNPs. Six cows were removed as they had a call rate below 0.9. After quality control, 39,269 SNPs remained for the genome-wide association analysis.

The statistical model used for estimation of (co)variance components included random and fixed effects, which were selected based on the Akaike information criteria obtained from the lme4 package in R software [20,21]. Variance components were estimated using the REML approach in airemlf90 software [22]. 

The final model was the following:

(1) $${\mathrm{CH}}_{4_{ijkl}}={\mathrm{LAC}}_{j}+\sum_{n=1}^{4}\beta_{n}{\mathrm{DIM}}_{kn}+{\mathrm{FYW}}_{l}+\sum_{n=1}^{3}animal_{in}{\mathrm{DIM}}_{kn}+\;\sum_{n=1}^{3}pe_{in}{\mathrm{DIM}}_{kn}+e_{ijkl}$$

in which ${\mathrm{CH}}_{4_{ijkl}}$ is the raw average daily CH_4_ concentration in ppm of the *i*th cow in the *j*th lactation, on the *k*th day in milk (DIM) over a range of 5th to 305th day, and the *l*th farm-year–week of the measurement; βn are fixed regression coefficients. Lactation (LAC) had two levels: 1 and 2+, for which records up to lactation 8 were included. The general lactation curve was modeled using third-order Legendre polynomials and random animal (*animal*), and permanent environment (*pe*) effects were modeled with second-order Legendre polynomials [23]. 

The covariance structure for model (1) was:

(2) $$var\left[\begin{array}{c} animal\\ pe\\ e \end{array}\right]=\left[\begin{array}{c} G⊗H\\ 0\\ 0 \end{array}\;\begin{array}{c} 0\\ P⊗I\\ 0 \end{array}\;\begin{array}{c} 0\\ 0\\ I\sigma_{e}^{2} \end{array}\right],$$

where **G** and **P** are covariance matrices of the random regression coefficients for animal and permanent environment effects, ⊗ is the Kronecker product, **H** is the combined pedigree and genomic relationship matrix, **I** is an identity matrix and $\sigma_{e}^{2}$ is the residual variance. The **G** matrix was defined as:

(3) $$G=\frac{ZZ\prime }{2\sum_{j}p_{j}\left(1-p_{j}\right)},$$

where Z contains genotypes adjusted with allele frequency and *p*_j_ is the allele frequency for marker *j* [24]. The **H** matrix was created as in [25,26]:

(4) $$H=\left(\begin{array}{cc} A_{11}-A_{12}A_{22}^{-1}+A_{12}A_{22}^{-1}GA_{22}^{-1}A_{21} & A_{12}A_{22}^{-1}G\\ GA_{22}^{-1}A_{21} & G \end{array}\right),$$

where subscripts 1 and 2 represent ungenotyped and genotyped animals, respectively, **A** is a pedigree matrix and **G** is a genomic relationship matrix. All three matrices were computed using the BLUPF90 suite of programs [22]. To investigate the existence of a possible population structure, we performed principal component analysis on the calculated **G** matrix. 

Some of the animals with phenotypes were not genotyped. Therefore, to utilize all the available information for estimating SNP effects, we used a single-step genomic BLUP (ssGBLUP) method that integrates phenotypes, genotypes, and pedigree information [25,26]. Genome-wide association studies were conducted with the BLUPF90 suite of programs [27]. The implemented algorithm uses the ssGBLUP procedure, which fits all the SNPs simultaneously and then obtains estimates of marker effects and their associated *p*-values from the estimated breeding values [28,29]. The main advantage of using ssBLUP for GWAS is the potential for using ungenotyped animals and the lack of need for using de-regressed phenotypes. The threshold for identifying significant SNPs was *p* ≤ 0.05; therefore, after Bonferroni correction to account for multiple testing (i.e., 0.05/39,269), we obtained a threshold of 5.9 on the -log10 scale. 

Next, we calculated the proportion of genetic variance explained by significant SNPs. In addition, we calculated the proportion of genetic variance explained by the moving windows of adjacent SNPs. We used two approaches to define the windows: in the first, window size was arbitrarily fixed to 50 SNPs, and in the second, window size was set according to the average length of the haplotype block. A haplotype block was required to have at least 95% of combinations of SNPs within a region in very high linkage disequilibrium [30]. As the average block length in the bovine genome is 69.7 +/− 7.7 kb, the window size was set to 70 kb. As a result, the number of SNPs grouped per window varied from 1 to 5 SNPs across the chromosomes. In the final step, potential candidate SNPs were checked in the Cow QTL database [31] for linkage to previously detected quantitative trait loci (QTLs) for CH_4_ production or other traits.

## 3. Results

### 3.1. CH_4_ Measurements

Variances of average daily CH_4_ ppm/d were 36,401 (CH_4_ ppm/d)^2^ for Farm 1 and 27,820 (CH_4_ ppm/d)^2^ for Farm 2 and differed significantly (Bartlett test *p*-value < 0.001). Variances of average daily CH_4_ g/d were 7789 (CH_4_ g/d)^2^ for Farm 1 and 10,962 (CH_4_ g/d)^2^ for Farm 2 and differed significantly (Bartlett test *p*-value < 0.001). Animals on Farm 1 and Farm 2 produced similar amounts of CH_4_ ppm/d (Table 1). Although Farm 2 had higher CH_4_ concentrations by about 12 ppm than Farm 1, differences between average CH_4_ ppm/d per farm were not statistically significant (Welch two-sample *t*-test). In the case of CH_4_ g/d (Table 1), Farm 2 had higher CH_4_ concentrations by 107 g and was significantly different from Farm 1 (*p*-value < 0.001, Welch two-sample *t*-test). 

### 3.2. Variance Components

**Figure 1:**
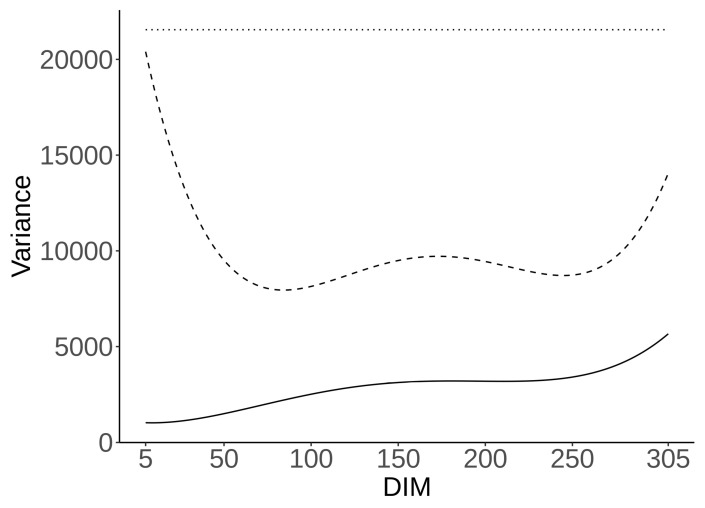
Residual (dotted line), permanent environment (dashed line), and genetic (solid line) variances (ppm/d)^2^ throughout lactation for CH_4_ ppm/d.

Genetic, permanent environment, and residual variances for CH_4_ ppm/d throughout lactation—estimated using model (1), identical to Pszczola et al. (2017) [7]—are presented in Figure 1. The genetic variance was, on average, 2928 (ppm/d)^2^ and systematically rose during lactation—reaching a maximum of 5967 (ppm/d)^2^ at the end of lactation. Permanent environment variance fluctuated during lactation, with an average of 9832 (ppm/d)^2^, a maximum of 20,466 (ppm/d)^2^ on the 5th DIM, a minimum of 7952 (ppm/d)^2^ on the 85th DIM, and 14,156 (ppm/d)^2^ on the 305th DIM. The residual variance was estimated at 21,568 (ppm/d)^2^. 

**Figure 2:**
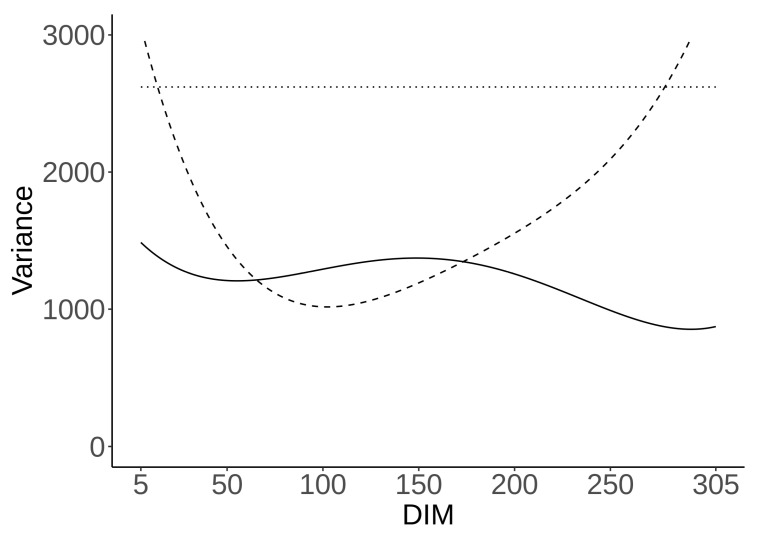
Residual (dotted line), permanent environment (dashed line), and genetic (solid line) variances (g/d)^2^ throughout lactation for CH_4_ g/d.

Genetic, permanent environment effect, and residual variances for CH_4_ g/d throughout lactation, estimated using model (1), are presented in Figure 2. The genetic variance fluctuated during lactation, with an average of 1199 (g/d)^2^, a maximum of 1486 (g/d)^2^ on the 5th DIM, and a minimum of 853 (g/d)^2^ on the 292nd DIM. Permanent environment variance also fluctuated with a mean of 1709 (g/d)^2^, a maximum of 3397 on the 305th DIM, and a minimum of 1016 on the 102nd DIM. The residual variance was estimated at 2620 (g/d)^2^.

### 3.3. Heritability Estimates

**Figure 3:**
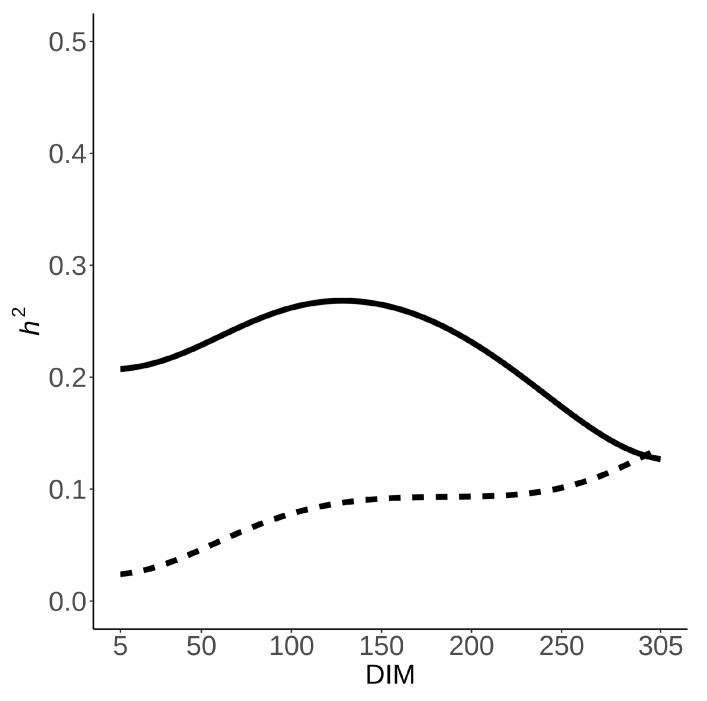
Heritability estimates for CH_4_ ppm/d (dashed) and CH_4_ g/d (solid) throughout lactation.

The heritability of CH_4_ ppm/d systematically rose during lactation (Figure 3) starting at 0 on the 5th DIM and reaching a maximum of 0.14 at the end. The average heritability was 0.085. The heritability of CH_4_ g/d (Figure 3) fluctuated with a mean of 0.22, a maximum of 0.26 on the 128th DIM, and a minimum of 0.13 on the 305th DIM. 

### 3.4. Genome-Wide Association Study

**Figure 4:**
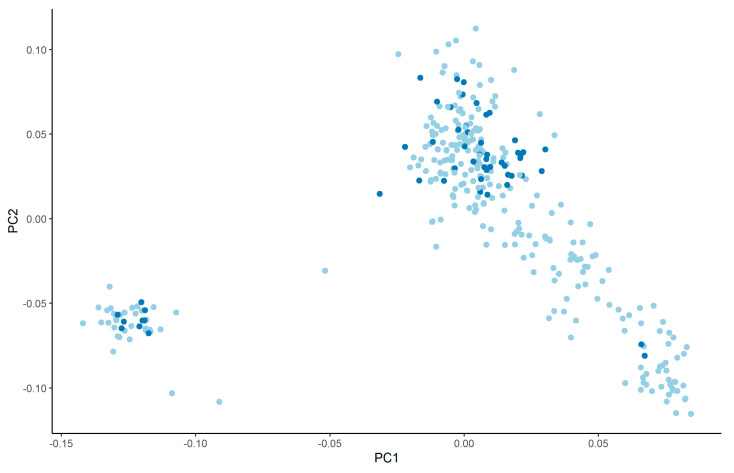
First (PC1) and second (PC2) principal components of the genomic relationship matrix with different colors for farms (Farm 1 = light, Farm 2 = dark).

**Figure 5:**
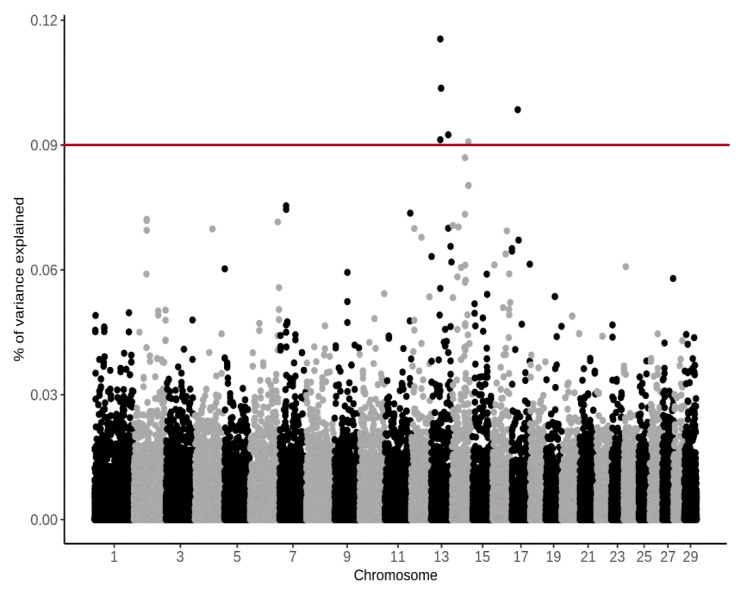
Manhattan plot for % of genetic variance explained by 50-SNP windows for CH4 ppm/d, with an arbitrary threshold line of 0.09%.

**Figure 6:**
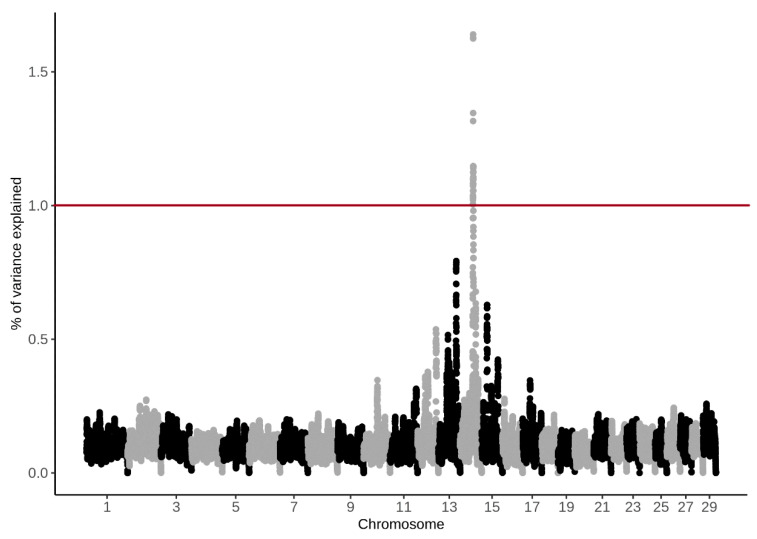
Manhattan plot for % of genetic variance explained by 50-SNP windows for CH4 g/d, with an arbitrary threshold line of 1%.

The principal component analyses showed two clusters (Figure 4), indicating the presence of a family structure. Animals from both farms were present in the two clusters. The obtained PC scores were small and explained 3.4% (PC1) and 2.5% of variance, respectively. *p*-values were obtained to identify candidate SNPs associated with CH_4_ ppm/d and with CH_4_ g/d, and we found that none of the tested SNPs was significantly associated with the phenotypes. To identify candidate regions, we evaluated SNPs in 70 kbp and 50 SNP windows for CH_4_ ppm/d and CH_4_ g/d. The analysis using 70 kbp windows did not detect any promising SNPs. However, the 50-SNP window approach detected potential candidate SNPs on BTA 14 which explained more than 0.09% of variance for CH_4_ ppm/d (Figure 5) and more than 1% of genetic variance for CH_4_ g/d (Figure 6). The discussed SNPs (Table 2) are located in the TRPS1 gene, which has been previously associated with milk fat yield, as evidenced by QTL:181825 [31].

## 4. Discussion

### 4.1. CH_4_ Measurements

The CH_4_ measurements varied across farms 1 and 2 for both CH_4_ ppm/d and CH_4_ g/d. According to Huhtanen et al. (2015) [32], potential sources of higher differences between farms may be sampling locations with different airflow patterns and cow variation in exhalation rate. Animals at Farm 2 were heavier (about 125 kg) and produced about 3 kg milk more per day (for more details see Table 1). This may indicate differences in feed intake or feed efficiency, although an investigation of this would require feed intake data, which were unavailable as the measurements were taken on commercial farms. 

### 4.2. Variance Components

Estimates of genetic variance for CH_4_ g/d were lower when compared to the study of Pszczola et al. (2017) [7], who used the same method and data, but no genotypes. Therefore, the use of genotypes lowered the estimates. Fluctuations of permanent environment variance in both traits and high levels of residual variance are probably as a result of unaccounted disturbances such as changes in the exhalation rate of the cows, head movement, or changes in the airflow around the feeding bin that could have been present during the measuring period [32,33]. 

### 4.3. Heritability Estimates

Pszczola et al. (2017) [7] reported an average heritability of 0.27 for CH_4_ g/d for pedigree-based analyses of 483 Polish Holstein-Friesian cows. In this current study, the addition of genotype data for 330 of the 483 animals resulted in a decrease in the heritability estimates, suggesting that the genetic influence on CH_4_ g/d may be lower than previously estimated. Lassen and Lovendahl [34] reported a heritability of 0.21 for Holstein-Friesian cattle, and heritability estimates for CH_4_ g/kg of dry matter intake (DMI) predicted from milk fat composition ranged between 0.12 and 0.44 [16]. A possible explanation for these differing results is the higher number of observations (e.g., Lassen and Lovendahl (2016) [13] used records collected from 3121 animals in their study). Direct comparison of CH_4_ heritability estimates from a range of studies requires traits that are comparable with one another. The CH_4_ g/kg of DMI in the study of van Engelen et al., 2015 [16] is predicted from milk fat composition; therefore, its heritability estimates depend on production parameters. Genomic studies based on CH_4_ g/kg of DMI could result in studying production parameters and not CH_4_ emission itself. An alternative could be studies based on direct CH_4_ measurements. The CH_4_ production expressed in CH_4_ g/d is calculated using a ratio of direct measurements of CH_4_ and CO_2_ multiplied by the predicted heat production, which is calculated using production parameters (i.e., fat–protein corrected milk), resulting in higher CH_4_ emission heritability estimates. Therefore, while the CH_4_ g/d is based on direct CH_4_ measurements, it is still to some extent associated with cow production traits. Lassen and Lovendahl (2016) [34] indicated a positive additive genetic correlation of 0.43 ± 0.10 between CH_4_ g/d and fat–protein corrected milk, which may have an impact on the results of the genetic studies on CH_4_ g/d. Van Engelen et al. (2017) [15] used log_10_-transformed CH_4_ ppm/d and obtained a heritability of 0.11. This was a similar phenotype to that of CH_4_ ppm/d presented in this current study, since it also is based only on direct CH_4_ measurements, without any additional traits involved in the calculations. Heritability estimates of both CH_4_ ppm/d and log_10_-transformed CH_4_ ppm/d are relatively low when compared to other CH_4_ phenotypes. While genetic studies based on CH_4_ ppm can remain unaffected by other traits, estimates of CH_4_ ppm/d or log_10_-transformed CH4 ppm/d are affected by environmental factors and animal behavior during the measuring time. The exhalation rate of the cow, head movement, or changes in the airflow around the feeding bin may affect CH_4_ ppm/d estimation and, as a result, may affect the estimation of CH_4_ ppm/d heritability [32,33]. It is, therefore, still unclear which phenotype is more reliable, and more studies are required in this field. 

### 4.4. Genome-Wide Association Study

The principal component analysis showed some stratification in the population; however, the magnitude of PC values was small, and both farms were represented in the two clusters (Figure 4). As shown by Mancin et al. [35], who analyzed the impact of different population structures on GWAS results, the level of stratification present in our data should have no influence on our analyses.

QTLs linked to the development of the digestive tract were identified by Pszczola et al. (2018) [9] using similar data, but with CH_4_ g/d phenotype and a Bayesian variable selection method [36]. In this study, we used single-step GWAS for post-analyses of ssBLUP, since it allows the inclusion of non-genotyped animals and thus provides more insight into the genetic architecture of the studied trait. The use of different methods on the same dataset also allows for the validation of previous results, as well as providing a reference point for the analysis of the new CH_4_ phenotype (i.e., CH_4_ ppm/d). In our current approach, none of the SNPs reported by Pszczola et al. (2018) [9] reappeared, which could indicate that Bayesian variable selection is more sensitive than the single-step GWAS approach. 

Several studies [37,38,39] used the GBLUP method for estimation of marker effects for GWAS and Aguilar et al. (2019) [28] indicated an empirical agreement between association studies on beef cattle in the literature and their findings based on single-step GWAS. Legarra et al. (2018) [40] also showed similarities between the results of the Bayesian approach and the GBLUP approach for GWAS studies. Pszczola et al. (2018) [9] used the Bayesian approach for the GWAS analyses of CH_4_ g/d, for which only genotyped animals could be used. In this current study, we decided to use ssGWAS, as it allowed us to include all the 483 phenotyped animals, of which only 330 were genotyped. A larger number of both genotyped and ungenotyped animals can improve the power of a GWAS analysis; however, in our case, it did not yield a higher number of significant SNPs as compared with Pszczola et al. (2018) [9]. 

In this study, we detected potentially novel SNPs for both CH_4_ ppm/d and CH_4_ g/d. The identification of the same SNPs in both traits—which express CH_4_ emission differently—may indicate a true association with CH_4_ emission. However, the found QTLs, similar to previous studies, explained only a small percent of the genetic variation— suggesting either the need for analysis on a larger dataset or a lack of SNPs with a large effect on the analyzed traits. Another possible reason for finding a small number of SNPs with significant effects on the investigated trait is the size of the SNP chip used. In this study we used the Illumina BovineSNP50 v2.0 BeadChip; possibly, a higher density chip would help to pin-point more significant SNPs.

## 5. Conclusions

This research aimed to study the genetic architecture of the CH_4_ ppm/day and CH_4_ g/day phenotypes. The obtained heritabilities of both traits indicated that CH_4_ emission is partially controlled by genes. While GWAS studies reported potentially novel SNPs associated with both CH_4_ phenotypes, all SNPs were associated with only one gene and explained only a small percent of the genetic variation. We conclude that both CH_4_ ppm/d and CH_4_ g/d are highly polygenic traits; thus, the identification of a specific gene or region will be challenging and will require large datasets. 

## Figures and Tables

**Table 1:** Average CH_4_ measurements, live weight, fat–protein corrected milk (FPCM) and number of records per farm.

	Farm 1		Farm 2	
	Mean	SD	Mean	SD
CH4 ppm/day	505	190	517	166
CH4 g/day	396	59.70	503	93.20
Live weight (kg)	544	115	669	73
FPCM (kg)	33.70	6.82	36.60	8.79
No. of records	31,179		3180	

**Table 2:** Candidate SNPs detected for both CH_4_ g/d and CH_4_ ppm/d, their position in base pairs, and percentage of genetic variance explained by them (%).

BTA	SNP Name	Position (bp)	(%) CH_4_ g/d	(%) CH_4_ ppm/d
14	BTB-00568855	48890778	1.62	1.19
14	Hapmap57707-rs29024913	48858864	1.31	1.01
14	UA-IFASA-9208	48726666	1.05	0.93
14	ARS-BFGL-NGS-45675	48679326	1.09	0.92

## Data Availability

Data available on the reasonable request to the corresponding author.
